# Supplementary material for: Are carbon emissions trading and green financial instruments synergistic? -Comprehensive quantitative research based on content analysis
Source: PLoS One. 2024 Mar 7;19(3):e0298601. doi: 10.1371/journal.pone.0298601 (PMC10919601; doi:10.1371/journal.pone.0298601)
Supplement: S1 Data — (ZIP) [file pone.0298601.s001.zip › Data/地级市Z_F工作报告.zip!/║╙─╧/╔╠╟≡╩╨/└┤╫╘╣1⁄2╓┌║┼í╛┬φ┐╦╩2╛▌═°í┐_2014.docx]

各位代表：

现在，我代表市人民政府，向大会报告政府工作，请予审议，并请各位政协委员和其他列席人员提出意见。

一、2013年工作回顾

刚刚过去的2013年，面对经济下行压力增大的严峻形势和艰巨繁重的改革发展稳定任务，在省委、省政府和市委的正确领导下，全市上下认真贯彻落实党的十八大和十八届二中、三中全会精神，抢抓承接产业转移的历史机遇，牢牢把握“持续求进，好中求快”的总基调，紧紧围绕主题主线，坚持以四项重点带全局，着力调整结构、着力扩大投资、着力改革开放、着力改善民生，各项工作取得新成绩。全市生产总值完成1538．2亿元，增长10．5％；固定资产投资完成1246．4亿元，增长24％；公共财政预算收入实现85．8亿元，增长22．3％；规模以上工业增加值完成539．5亿元，增长16．2％；社会消费品零售总额完成623．4亿元，增长14．2％；城镇居民人均可支配收入20250元，农民人均纯收入7100元，分别增长10．5％和10．8％；居民消费价格上涨2．3％；节能减排完成省定目标任务，全市经济社会发展继续保持了好的趋势、好的态势和好的气势。

一年来，我们主要抓了以下七个方面的工作：

（一）抓好经济运行调节，推动经济平稳较快增长。面对经济下行的困难形势，我们未雨绸缪，以稳经济、保增长为重点，狠抓经济运行监测和综合协调，强化重点区域、重点行业、重点企业运行调节，优化生产要素配置，组织开展了百日攻坚行动，有效推动签约项目促开工、在建项目促进度、建成项目促达产、运营项目促增效。规模以上工业增加值增速一直在16％～16．8％的区间运行，利润增速从上半年的0．08％回升至全年的13％；工业用电量增速从年初的－0．4％回升至全年的7．4％，全市工业经济运行实现了稳中趋升、稳中有进的良好态势。积极组织“金融服务进产业集聚区”活动，为企业新增贷款130亿元，市外融资93亿元，创历史新高，有效保证了各类企业的资金需求。大力促进产销对接，组织参加中博会，成功举办了电动车、三轮车及零部件展览会，提升了商丘制冷产业和电动车品牌影响力，扩大了市场份额。深入开展企业服务活动，在全省率先建成“商丘市中小企业服务中心”，着力拓市场、破瓶颈、解难题，促进了民营经济、中小企业创业创新、转型升级和健康发展。商贸物流业快速发展，全市年交易额超10亿元的市场达到12家，继农产品中心批发市场之后，中原车城成为全市第2家销售收入超百亿元的市场，各类市场主体总量突破20万户，居全省第3位。

（二）做好承接产业转移，促进产业集群集聚发展。以承接产业转移示范市建设为抓手，围绕主导产业，进一步内引外联，吸引带动配套和相关企业跟进转移，产业集群化态势已经显现。民权县制冷产业形成了年产冰箱冷柜1200万台、压缩机300万台、冷藏保温车1．5万辆的生产能力。柘城县金刚石产业形成了从单晶合成到微粉加工再到系列制品生产较为完善的链条。夏邑县纺织产业规模不断壮大，形成了纺织、印染、服装加工为一体的产业链。虞城县打造以钢卷尺为主的五金电子特色主导产业，年产五金工具1．2亿只、钢卷尺10亿只以上。睢县电子信息产业依托富士康实训工厂和培训基地，年产手机配件1亿多片。宁陵县农资化工产业已入驻史丹利、嘉施利、君信等一批龙头企业，年生产能力达到550万吨。睢阳区纺织针织服装、梁园区医药制造、开发区电动车和环保设备产业，都已形成重要的生产能力，具备了区域竞争优势。目前全市各个产业集聚区的主导产业更加清晰，都形成了自己的产业集群，主导产业营业收入占整个产业集聚区规模以上工业营业收入比重达50％以上。

（三）坚持四项重点工作，保持全局向好发展态势。坚持把产业集聚区、招商引资、项目建设、新型城镇化四项重点作为战略性举措来抓，以重点突破带动全局发展。产业集群区建设水平不断提升。突出“三规合一”、“四集一转”、产城融合，基础设施更加完善，综合承接能力更加凸显。全市产业集聚区完成固定资产投资850亿元以上，占全市固定资产投资总量的70％；实现规模以上工业增加值250亿元以上，占全市规模以上工业增加值近60％；税收收入40亿元以上，带动41万人就业。在2013年全省产业集聚区观摩点评活动中，我市获得黄淮四市第1名；3个产业集聚区进入全省第一批专业示范产业集聚区行列，总数居全省第1位。开放招商力度不断加大。坚持把开放招商作为推动经济发展的重要抓手，突出招大引强，阿诗丹顿电器、格力顿汉、泛亚电缆等一批知名企业和项目落户商丘。与阿里巴巴（中国）网络技术有限公司、中国联通河南省分公司签署了合作协议，共建阿里巴巴商丘产业带，通过电子商务平台打造全新的网上物流中心和集散地。全市实际利用省外资金完成470．4亿元，总量、增速和完成比例均居全省第3位；引进境外资金2．77亿美元，增长12．4％，超额完成省定目标任务。新型城镇化引领作用明显增强。中心城区建设实现了重大突破，投资规模创历史新高。启动了城市总体规划修编，中心城区建成区面积达到116平方公里，人口达到116万人，城镇化率达到43．8％。商丘新区共引进项目49个，总投资579．6亿元，白云仓储、亿丰物流、大一机电等项目开工建设，中原佳海国际商贸城、义乌国际商贸城、富商新能源产业园等一批大项目相继签约。

商务中心区建设加快推进，宋城路、中州路和睢阳路3条主干道基本完工，日月湖引黄调蓄工程正在加紧施工。郑徐高铁建设进展顺利，商合杭高铁项目已获国家批准。商丘古城开发保护取得突破性进展，国家开发银行支持古城棚户区改造的21亿元长期贷款已获批准。深入开展“六城”联创活动，城市建设管理水平不断提高。各县（区）紧紧抓住商务中心区和特色商业区建设，着力提升承接产业转移、服务经济发展的能力，实现了县域城市的快速发展。中心镇、特色镇和新农村建设得到长足发展。全市启动旧城、棚户区、城中村改造项目110个，完成房屋征收753万平方米、新开工建设安置房370．3万平方米，房地产开发施工面积553．4万平方米。重点项目建设取得明显成效。组织实施了重点项目“百日攻坚活动”、“战酷暑强投资促进度活动”，项目投资拉动作用进一步增强。全市安排市管重点项目253个，全年完成投资715亿元，占年度计划的136％，较上年增长32％，其中省管重点项目完成投资112亿元，占年度计划的160％。重点项目的持续健康发展，既为保增长提供了保障，又为商丘的未来奠定了基础。

（四）强化农业基础地位，加快新型农业现代化进程。狠抓粮食核心区建设，新建成“百千万”高标准粮田126万亩，全市粮食产量达到128．8亿斤，实现“十连增”，再次荣获全国粮食生产先进市。大力发展果蔬、花卉等高效农业，加快畜牧标准化养殖小区建设，继续发展水产养殖业，提高优质、特色农畜产品比重。加快农业产业化集群培育，发展市级以上农业产业化重点龙头企业270家，其中国家级4家、省级53家，农业产业化经营组织4118家，科迪乳业、诚实人面业等9个农业产业化集群被省政府认定为2013年河南省农业产业化集群。狠抓农业生产条件改善，全市共投入水利建设资金16．9亿元，不断完善灌排体系，灌溉面积达到875．7万亩，抗御自然灾害和旱涝保丰收能力进一步增强。解决农村87万人饮水安全问题。全市农机总动力达1176万千瓦，农作物综合机械水平达到82％。林业生态建设成绩显著，林木覆盖率达到28．6％，完成林业生态建设9．8万亩，任务完成率位居全省第1位。

（五）突出改革创新驱动，不断增强经济发展活力。把改革创新作为重大战略加快推进，持续破解要素瓶颈制约，切实增强发展活力。创新粮食主产区土地流转机制，稳步推进土地规模经营，全市农村土地并块面积达870万亩。农业部在我市召开了由12个省农业部门参加的“互换并地”现场会，推广我市“小块并大块”工作经验。创新土地资源保障机制，积极抓好土地综合整治、城乡建设用地增减挂钩、耕地占补平衡和争取人地挂钩试点工作，申报各类建设用地报件83批次、5．3万亩，创造了我市年度申报土地的新纪录，有效保障了工业化、城镇化用地。创新资金保障机制，完善金融机构考核奖励办法，引导金融机构对经济特别是小微企业的支持，小微企业贷款增幅居全省第1位。创新人力资源保障机制，加快发展职业教育和劳动技能培训，鼓励外出务工农民回乡就业创业，更多的劳动者实现了在家门口就业。大力推进科技自主创新体系建设，建成和在筹建的国家级质检中心3个、省级质检中心7个，新增省级工程技术研究中心8个，高新技术企业14家，国家级科技企业孵化器1个，河南省院士工作站1个，柘城县产业集聚区被科技部认定为国家超硬材料及制品高新技术产业化基地。

（六）注重保障改善民生，扎实推进和谐商丘建设。继续加大投入，办好省定“十项重点民生工程”和市定“十件实事”，全市各项民生支出达245亿元，占公共财政预算支出的77．7％。促进就业再就业工作，着重做好大学生、弱势群体、“4050”人员等重点人群的就业。全年下岗失业人员再就业2．97万人，就业困难人员就业1．16万人，均超额完成年度目标任务。扎实推进社会保障工作，城乡居民养老、医疗、工伤和失业保险覆盖率均达95％以上，城乡低保、五保供养标准进一步提高。全年归集住房公积金10．5亿元，通过住房公积金解决了1．3万户城镇中低收入家庭住房问题。完成90个贫困村整村推进，12．7万贫困人口实现脱贫。投入资金19．5亿元，用于保障性安居工程建设，圆满完成省定目标。高度重视教育事业，学前教育三年行动计划圆满完成，新建、改扩建幼儿园565所，改造农村义务教育薄弱学校116所、县镇普通高中5所，促进教育资源均衡配置。免费向贫困县义务教育阶段23万名学生提供营养餐，免费向城市义务教育阶段学生提供教科书，基本解决了农民工随迁子女在城市接受义务教育问题，九年义务教育巩固率达到92％，高中阶段教育毛入学率达到90．2％，均比上年有所提高。支持职业教育品牌示范院校和特色院校建设。深入实施文化惠民工程，全市博物馆、图书馆、群艺馆（文化馆）、乡镇综合文化站等公益性文化设施均已免费开放，大运河商丘段申遗工作扎实推进。旅游产业健康快速发展，旅游人数和旅游收入分别增长13．5％和15．6％。广泛开展全民健身运动，大力开展竞技体育，在第12届全国运动会上取得了优异成绩。强化疫情监测分析，做好手足口病、甲型H7N9流感、结核病等重大传染病防治工作。稳定低生育水平，人口自然增长率控制在5．2％。左右。不断加强民族和宗教工作，促进了民族团结进步和经济发展。注重源头预防，积极推行社会稳定风险评估，评估重大决策事项43起，有效预防和减少了因决策不当引发的矛盾纠纷。落实领导干部接访处访各项规定，畅通信访渠道，解决实际问题，市县党政领导接待信访案件1128件，办结率95％。加强平安建设，构建和谐商丘，社会治安防控体系不断完善，防控水平全面提升，打防违法犯罪成效显著，社会大局持续平稳，人民群众安全感和满意度进一步提升。全面落实安全生产责任制，突出抓好食品安全监管，全年未发生重特大安全事故。

（七）加强民主法治建设，不断提高社会文明程度。一年来，我们自觉接受人大依法监督、政协民主监督和社会各界监督，认真执行市人大及其常委会决议，依法向市人大常委会报告工作，定期向市政协通报工作。745件人大代表建议和政协委员提案如期办复完毕，办理质量不断提高。出台了重大行政决策程序规定，坚持依法、公开、科学、民主、效率的原则，遵循公众参与、专家咨询、风险评估、部门论证、合法性审查、集体讨论决定的程序，不断增强行政决策的科学性、合理性。加强规范性文件的审核清理和重大合同的审核备案，政府依法行政水平不断提升。依法推进政府信息公开，深入实施政风行风评议，政府自身建设取得明显成效。深入开展法制宣传教育，全面落实“六五”普法规划，全民法律素质进一步提高。我市顺利通过省级文明城市先进市考评、省级卫生城市复核检查验收，成功创建省级双拥模范城，国家森林城市规划编制顺利通过国家林业局专家评审，在打造宜居、宜业城市方面取得了初步成效。实施公民道德素质提升工程，大力弘扬“一言九鼎，实干兴商”的商丘城市精神，广泛宣传“商丘好人”群体，全市共有50人入选“中国好人榜”，13人荣登“中国好人榜”，5人获得全国、全省道德模范提名，商丘人可交、干部素质高、地方风气正、发展环境好的软实力不断增强，美誉度日益提高。

加强国防教育和国防后备力量建设，积极推进军民融合式发展，驻商解放军、武警部队和民兵预备役人员在商丘经济社会发展中作出了重要贡献，巩固了军政军民团结的良好局面。外事侨务、对台工作进一步加强，统计、供销、邮政、盐业、烟草、史志、地震、人民防空、档案管理、社科研究、青少年、妇女儿童、老龄、残疾人和慈善事业等工作取得了新的成绩。政府性债务审计工作顺利完成，第三次经济普查工作进展顺利，对口援疆工作圆满完成。

各位代表！

这些成绩的取得，得益于省委、省政府和市委的正确领导，得益于市人大、市政协的监督支持，得益于社会各界的大力支持，更凝聚着全市人民的智慧和汗水。在此，我代表市人民政府，向各位人大代表、政协委员，向各位老领导、老同志，向中国人民解放军驻商部队和公安干警、武警消防官兵，向各民主党派、人民团体、工商联和无党派人士，致以崇高的敬意！向所有为商丘建设发展作出贡献的同志们、朋友们，表示衷心的感谢！

在肯定成绩的同时，我们也清醒地看到，规模以上工业增加值、社会消费品零售总额增速虽然总体上高于全省平均水平，均居全省第3位，但由于主客观因素的影响，略低于年初确定的预期目标。同时，全市经济社会发展还面临很多困难和挑战，政府工作还存在不少问题和差距。一是经济基础仍然薄弱，经济总量偏小，发展质量不高，经济增长内生动力不强，一些企业活力仍显不足。二是在经济结构方面，服务业占比低，消费需求不足，煤电铝等重点行业发展形势不容乐观，结构调整仍然面临较大压力。三是土地、资金、用工等生产要素制约明显，环境容量趋紧，出境断面的水容量几乎饱和，大气排放容量空间不大，发展新的产业将面临很大的环境制约，可持续发展、绿色发展、低碳发展的任务还很重。四是在政府工作中，还存在服务不优、效率不高的现象，少数部门还存在门难进、脸难看、事难办的问题，极少数部门还存在着“吃、拿、卡、要”等现象。对此，我们一定高度重视，采取有效措施加以解决。

二、 2014年政府工作总体要求和目标任务

今年是全面贯彻落实党的十八届三中全会精神、全面深化改革的第一年，也是完成“十二五”规划目标任务的关键一年。今年政府工作的总体要求是：以党的十八大和十八届二中、三中全会精神为指导，认真贯彻落实省委经济工作会议、省“两会”和市委经济工作会议精神，紧紧围绕构筑中原经济区东部战略支撑总目标，坚持“持续求进、好中求快”的总基调，把改革创新贯穿于经济社会发展的各个领域各个环节，抢抓粮食生产核心区、中原经济区、郑州航空港经济综合实验区三大国家战略规划机遇，扭住发展不放松，坚持以四项重点带全局、四区发展求突破，着力优化结构，着力培育优势，着力改革创新，着力扩大投资，着力改善民生，以调促增、以变促进、以转促好、以改促活，扩大承接产业转移示范市建设成效，切实提高经济发展质量和效益，促进经济持续较快健康发展、社会大局和谐稳定。

今年经济社会发展主要预期目标是：在提高质量和效益的基础上，全市生产总值增速在10％以上；公共财政预算收入增速高于生产总值和全省平均增速2个百分点以上；全社会固定资产投资增长22％；社会消费品零售总额增长14％；外贸出口增长10％；实际利用外商直接投资增长10％；城镇居民人均可支配收入增长9％，农民人均纯收入增长10％；人口自然增长率控制在6．5％。以内；居民消费价格涨幅控制在3．5％左右；节能减排完成省定目标。

今年经济增长预期目标定为10％以上，体现了中央和省委、市委经济工作会议精神，是根据我市发展需要和可能制定的，就是要引导各方把主要精力和工作重心放到加快转变发展方式和调经济结构上来，以实现更长时期、更高水平、更好质量发展。

三、 2014年重点工作

今年，着重做好以下八个方面的工作：

（一）推动结构战略性调整，构建现代产业发展体系。积极打造产业发展平台，大力发展工业和现代服务业，增加二三产业在地区生产总值中的比重，推动产业结构进一步优化和提升。加快完善工业体系。围绕打造“一谷三城六基地五中心”的目标，大力发展工业，推动产业结构优化升级，构建现代新型工业体系。改造提升传统产业，对化工、冶金、建材、纺织服装等传统产业，综合运用财税支持、技术改造、兼并重组等手段，强化项目引进、技术改造和精深加工，推动传统产业健康、快速发展。不断壮大优势产业，对食品加工、制冷设备等产业，着眼于跨越发展，食品工业主要巩固提升面及面制品加工、畜禽屠宰加工、酿酒等传统食品产业，积极培育乳品加工、果品饮料、速冻休闲食品等新兴食品产业，制冷工业主要完善制冷研发、制冷检测、冷链物流等配套产业，形成专业化分工、产业化协作相对完备的产业体系。培育高成长性产业，对新材料、生物医药、电子信息、新能源、现代家居、环保设备等新兴产业，着眼于创新发展，重点开发高性能纤维产品和复合材料、金刚石微粉及制品、生物医药、集成电路、电子元件等产品，提升新兴产业产值比重和发展权重。大力发展第三产业。把发展第三产业作为促进经济结构优化升级的战略性举措来抓，加快发展市场平台建设。建设物流节点城市，完善豫东综合物流集聚区基础设施，加快推进白云仓储物流、亿丰国际商业博览城、商丘花木交易市场等项目建设进度。依托商务中心区建设，加快建设现代化的商务景观、会展等设施，培育楼宇经济，发展金融、中介、研发等现代服务业。以古城保护开发为契机，培育壮大文化旅游产业，积极发展文化观光游、生态休闲游和乡村体验游等新型旅游业态，加快旅游资源优势向产业优势转化。以梁园区市场建设为重点，推进红星美凯龙、丹尼斯二期、大商新玛特二期、大润发、中濠国际广场等项目建设，实施中原车城升级改造，加快发展生活性服务业。大力发展数字城市、电子政务、电子商务等信息产业，充分发挥阿里巴巴（商丘）产业带的作用，鼓励企业拓展网上营销渠道。各县（区）以中心商务区和特色商业区为载体，以服务产业发展为重点，加快发展生产性服务业，以服务人口转移、促进人口聚集为重点，加快发展生活性服务业。积极发展信息、科技、咨询、第三方物流、家居养老等新型服务业态。加强发展载体建设。围绕加快二三产业发展步伐，重点推进产业集聚区、中心商务区和特色商业区建设。产业集聚区要加强软硬件环境建设，突出产城互动，把集聚区内道路、通信、水电、污水垃圾处理、公租房等列入城市基础设施建设计划，统筹规划建设创新孵化、综合服务、检验检测、仓储物流等公共服务设施，提高产业集聚区综合承载能力。全市10个产业集聚区力争完成固定资产投资1000亿元，实现主营业务收入1700亿元，工业增加值260亿元，形成主营业务收入超200亿元的产业集聚区5个。结合省里出台的产业集聚区考评办法，强力推进升级晋位和示范创建工作，每个产业集聚区瞄准1～2个方向开展创建，努力实现在省内排位前移，争取更多的产业集聚区进入全省示范行列。中心商务区和特色商业区加强基础设施建设，完善功能，做好配套，提高承接现代服务业能力。

（二）加快粮食核心区建设，提升农业现代化水平。强化粮食生产安全，是粮食生产核心区义不容辞的政治责任，也是稳固农业基础地位、加快推进新型农业现代化的需要。实施好高标准粮田“百千万”工程。加强农田水利、电网改造、农业综合开发等项目建设，积极开展土地综合整治和高标准粮田建设，实施好中小型水库除险加固、中小河流治理、干支渠系配套完善等工程，全年新建高标准粮田89万亩。加强农业服务体系建设，打造农业技术、农机、供求、流通等农业信息服务平台，强化农业生产过程中的社会化服务。大力推广农业科技实用技术，促进粮食高产创建，确保全年粮食产量稳定在120亿斤以上。实施好农业产业化集群工程。重点培育科迪乳业、诚实人面业、贵友生猪、中州食用菌、丰太饮品等9个区域性优势和特色产业集群，力争建设一批规模化、标准化、集约化的种植和养殖原料生产基地。延长产业链条，新增5个以上省级重点龙头企业、10个以上市级重点龙头企业，力争培育一个销售收入超100亿元、4个超20亿元的农业产业化集群，实现食品工业销售收入600亿元以上。实施好精品农业工程。积极推进农业产业结构调整，按照稳猪、增禽，突出发展奶牛、肉牛产业的思路，继续抓好黄河故道新增1万头奶牛养殖等项目建设。搞好农业产品结构调整，城市近郊大力发展设施农业、休闲观光和生态农业，做大做强宁陵酥梨、虞城惠楼山药、夏邑食用菌、柘城三樱椒、梁园区草莓、睢阳区和睢县烟叶等特色农业。培育专业大户、家庭农场、专业合作社、农业产业化龙头企业等新型农业经营主体，加快推进农村土地“小块并大块”工作，促进农村土地合规有序流转、规模化经营，实现农业稳定增效、农民持续增收、农村改革发展。实施好扶贫开发工程。编制实施好我市大别山区域扶贫攻坚与小康建设规划，完成90个贫困村的整村推进工作，实现11．7万农村贫困人口稳定脱贫。

（三）强化三级城镇体系建设，推进新型城镇化快速发展。全面贯彻落实中央城镇化工作会议精神，按照省新型城镇化指导意见和三年行动计划的要求，坚持以人为本、优化布局，生态文明、传承文化，遵循规律、因势利导，积极稳妥、规范有序地推进新型城镇化。提升中心城区辐射带动能力。优化中心城区空间布局，完善功能，加快推进商丘市总体规划修编工作。以郑徐高铁商丘站、古城保护开发和商务中心区建设三大项目为抓手，带动城市基础设施建设加快发展。全面开工郑徐高铁商丘站整体建设工程，基本完成郑徐高铁主线线下工程，开工建设凯旋路和归德路穿跨工程，统筹做好站前广场整体开发，力争完成投资100亿元。古城保护开发计划投资40亿元，完成安置用地的规划选址和一期工程80万平方米主体建设，启动东南城湖开挖清淤和应天书院、归德府衙恢复建设项目。商务中心区完成宋城路、中州路、睢阳路3条主干道的综合配套设施建设，实施秦商、汉商等8条次干道建设；充分利用土地、城投债等融资模式，力争基础设施建设融资25亿元以上；加强与建业、中信、华强等企业合作，力争入驻项目100个以上，实现当年投资50亿元以上。商丘新区在完善总体规划和专项规划的基础上，重点推进105国道、郑庄、平台互通式立交桥“两桥一路”和胜利路、310国道城区段建设。城区道路重点抓好神火大道（北海路～华商大道）拓宽改造、睢阳大道大修改造等工程。城区园林绿化计划投资1．7亿元，重点抓好汉梁文化园、商丘植物园、华夏游乐园和部分道路的绿化建设，对京港绿地、文化绿地、金世纪广场、商都文化广场等进行升级改造，为我市2015年顺利通过国家园林城市复查奠定基础。持续推进内河水系综合治理，计划投资3亿元，继续实施包河、运河、蔡河、万堤河、忠民河、康林河等6条城区内河部分河段的综合治理工程。加快旧城、城中村、棚户区改造步伐，改善老城区居民生活环境。继续推进“六城”联创工作，提升城市建设管理水平。大力发展城区经济，依托商丘新区、梁园和睢阳产业集聚区、开发区、豫东综合物流集聚区以及食品工业园区，积极引进工业项目，加快新材料、环保专用设备、电动车等专业园区建设，力争完成投资300亿元以上，培育和完善城区工业体系。加大城区现代服务业和商业业态建设，进一步提升中心城区的现代服务业水平。继续稳定房地产业发展，完成房地产开发投资52亿元。强化县级城市集聚产业实现产城互动能力。按照每个县城30～50万人口规模，做好城镇化发展规划，加强县城基础设施和公共服务设施建设。以商务中心区和特色商业区建设为抓手，以服务生产、生活为主要目的，完善基础配套功能，增强对产业项目的吸引力，力争完成固定资产投资70亿元，其中基础设施投资20亿元，力争每个商务中心区、特色商业区引进1个以上知名商业地产企业或投资5亿元以上的龙头项目。加强产业集聚区基础配套设施建设，实现产业集聚区与县域城区公共设施的有效对接，加快商务办公、职工宿舍、文化场所、商业购物、公共食堂等生产性配套设施建设，为产业发展提供有效支撑。增强特色乡镇吸纳农村人口转移就业能力。对有特色和产业基础的中心镇、具有资源禀赋和区位优势的小城镇、有条件的中心村，做好配套设施建设，增强产业支撑和辐射带动作用，支持中心镇逐步发展形成一批5万～10万人以上的小城市。突出培育夏邑会亭镇、虞城站集镇、宁陵张弓镇等工业型乡镇，睢阳郭村镇、柘城胡襄镇等商贸型乡镇，梁园刘口乡、民权北关镇等旅游型乡镇，夏邑车站镇、梁园双八镇等特色农业型乡镇，培育一批文化旅游、商贸物流、资源加工、交通节点等专业特色镇，带动农村人口就近就业，有序转移。稳步推进新农村建设。以县为单元，按照产业、新农村、土地、公共服务和生态规划“五规合一”的要求，推进新农村规划编制。科学确定村庄布局和规模，配套建设基础设施和公共服务设施，全面改善农村生产生活条件。

（四）实施开放带动战略，加快外向型经济发展步伐。继续实施开放带动战略，持续抓好招商引资，加快形成全方位、多层次、宽领域的对外开放格局。抓重点招商。突出重点招商区域，主攻长三角、珠三角、闽浙粤、环渤海等区域，分类实施专业招商、专题招商、小分队招商等方式，提高招商引资成功率。突出主导产业招商，重点引进制冷、食品、纺织服装、新材料、生物医药等产业。突出招大引强，瞄准国内外500强企业、著名品牌、上市公司、行业龙头，重点引进跨国集团、央企、省企、大型民企以及配套服务的中小型企业，全年实现引进省外资金500亿元以上。抓区域对接。抢抓郑州航空港综合经济实验区、上海自由贸易实验区等机遇，发挥区位、交通、资源和产业优势，在基础设施、产业发展、经贸交流、技术合作等领域主动对接，努力扩大与郑汴洛、长三角等区域的战略合作。抓项目推介。办好2014年国际华商节，利用第九届中博会、第十八届投洽会、第十六届高交会等重大招商活动，做好我市项目的包装和推介，主招产业链缺失环节、核心技术、配套产品。组织我市企业和项目“走出去”，积极参与省政府组织的港澳台、新加坡、韩国等境外招商引资活动，多渠道、宽领域引进境外资金。抓对外合作。抓好重点企业、重点商品的供货转自营出口工作，依托虞城县汇丰保税仓库，扩大进口规模，壮大进出口总量。发展国际友好城市和周边国家贸易往来，扩大对外经济合作，努力以对外直接投资、开发合作项目、对外承包工程等带动设备出口和外派劳务。抓好开放平台建设，加快推进保税物流中心方案编制、上报等工作。

（五）加强基础支撑能力建设，培育加快发展竞争优势。把重大项目建设作为加强基础支撑能力的重要抓手，今年计划安排省市重点项目245个，总投资2042亿元，当年计划投资748亿元。强化基础设施支撑。以建设中原经济区综合交通枢纽为目标，重点实施好郑徐客运专线、连霍高速公路拓宽改造、商登高速、沱浍河航运、商丘高铁核心区市政配套一期工程，推进商合杭客运专线、商丘机场等项目前期工作进度，争取年内开工建设，实施长江路东延工程，构建以商丘为中心，放射状、网格化、立体式综合交通运输网络。做好电网科学规划，加快市区110千伏潘洼扩建输变电、220千伏柘城输变电等工程建设，有序提升电网智能化水平。完善信息基础设施，推进4G网络建设，发展现代信息技术产业，促进工业化和信息化深度融合。强化产业基础支撑。推动柘城金锐金刚石产业园、宁陵复合肥生产基地、梁园安踏产业园和国药基地等尽快建成投产，发展壮大民权制冷、夏邑和睢阳区纺织服装、虞城五金电子、开发区电动车和环保专用设备、睢县电子配件和制鞋等产业集群，打响品牌，提升质量，提高集聚发展水平。优化能源结构和布局，加快推进神火集团2×35万千瓦热电联产机组、国电民权电厂二期2×100万千瓦机组项目前期工作，实施西气东输豫东支线二期工程等一批电源、电网、煤矿、油气管道、新能源项目。积极推进中石油千万吨炼油厂前期工作，争取列入国家“十三五”规划。同时，谋划、储备一批事关商丘长远发展的重大项目，适度超前发展环保、能源、新材料等基础产业，增强经济发展后劲。强化科技创新支撑。把科技创新摆在发展全局的核心位置，重点围绕高成长性产业、传统优势产业和战略性新兴产业，采取政府引导、以奖代补等措施，鼓励企业、院校和科研单位建立研发中心、科技孵化器、院士工作站、产业集聚区创新服务平台等，构建以企业为主体、市场为导向、产学研相结合的技术创新体系。加大科技创新投入，增强企业技术创新能力。推进标准化和品牌带动战略，增强企业和产品竞争力。强化人力资源支撑。实施全民技能振兴工程、职业教育攻坚工程、“阳光工程”和“雨露计划”，培育高素质技能型人才。加大企业家队伍和企业经营管理人才的培训，完善吸引和留住人才的体制机制，加大高层次紧缺人才引进力度，积极培育创业人才队伍。深化选人用人制度改革，建立激励竞争机制，优化干事创业环境，吸引高端人才集聚。

（六）深入推进全面改革工作，完善科学发展体制机制。按照中央和省统一部署，以经济体制改革为重点，积极稳妥推进我市的改革工作，以改革促发展促创新，破解体制机制难题。激发市场主体活力。正确处理政府和市场的关系，建设和维护统一开放、竞争有序的市场体系，真正使市场在资源配置中起决定性作用。深化国有企业股份制、公司制改革和企业内部改革，完善法人治理结构，加强国有资产监管，逐步建立国有资产全覆盖监管体系，提高国有资本运营效益。支持非公有制经济发展，鼓励非公有制企业参与国有企业改革，鼓励有条件的民营企业建立现代企业制度。认真落实公司注册资本登记制度改革，减少前置审批事项，实施中小微企业成长工程。深入开展与央企合作战略，积极参加“中央企业中原行”活动，主动对接央企和省管大型国有及国有控股企业，积极争取我市与央企合作实质性项目落地。扎实推进财税改革。完善全面规范、公开透明的预算制度，建立跨年度预算平衡机制，清理和规范重点支出同财政收支增幅或生产总值挂钩事项。清理、整合专项转移支付项目，提高财政资金使用效益。清理规范税收优惠政策，全面落实国家“营改增”、资源税从价计征等改革政策。加强预算绩效管理，从严控制一般性支出，确保“三公经费”只减不增。加强政府债务管理，有效防范和化解财政风险。深化金融改革。充分利用多层次资本市场、多种金融产品，推进一批企业上市融资和挂牌转让股份，落实好永续债券、县城投集合债券和债贷统筹试点，积极引进股份制银行。加快地方金融机构改革，积极配合中原银行组建工作，研究探索农村金融改革创新，支持符合条件的县域农信社组建为农商行，加快村镇银行组建步伐，探索建立城市基础设施、住宅政策性金融机构，用活民间资本，支持具备条件的民间资本依法发起设立金融机构。全面建立风险补偿机制，优化小微企业融资环境，规范民间融资，打击非法集资，防范和化解金融风险。加快户籍制度改革。有序放开商丘市区落户限制，全面放开县城和建制镇落户限制，逐步提高户籍人口城镇化水平。推行居住证制度，建立以居住证为依据的基本公共服务提供机制。加快农业转移人口市民化，重点解决一批已进城就业定居的农民工落户、一批城中村居民成建制转户。深化土地管理制度改革。探索建立兼顾国家、集体、个人的土地增值收益分配机制，力争年底前完成农村宅基地确权登记和颁证工作。探索宅基地有偿退出机制，积极争取开展宅基地制度改革试点，研究在符合规划和用途管制前提下，抓好农村集体经营性建设用地进入市场流转试点。积极用好城乡建设用地增减挂钩政策，争取人地挂钩政策试点，增强用地保障。探索实行租让结合、分阶段出让的工业用地供应制度。严守耕地保护红线，加大土地收购储备力度，严格土地供应管理，盘活存量，优化结构，提高建设用地利用效率。

（七）提高生态文明建设水平，建设生态商丘美丽商丘。建设生态文明，关系人民福祉，关系经济发展的质量和效益，把生态文明建设融入经济社会发展的全过程，推动绿色发展、循环发展、低碳发展，增强可持续发展能力。强力推进节能减排。认真实施蓝天工程，积极推进重点行业脱硫脱硝除尘改造、城市燃煤锅炉改造、机动车尾气治理、城市施工和道路扬尘防治、秸秆焚烧整治，完成国电民权电厂脱硝工作，认真实施碧水工程，做好产业集聚区污水处理厂及配套管网建设，力争6月前全部通过验收。严格污染物排放标准，大力发展节能环保产业，进一步减少污染排放，确保大沙河、包河等5条河流出境断面稳定达标。认真实施乡村清洁工程，开展农村连片环境综合整治和农作物秸秆综合利用，推进“美丽商丘·整洁村镇”活动深入开展，改善农村人居环境质量。大力发展循环经济。加快推进循环经济试点建设，实施一批重大项目和示范工程，提升循环经济发展水平，建设工农业复合型循环经济。依托优势资源，以产业集聚区和重点企业为载体，重点打造有色、化工、生态农业、环保设备和环保产业以及再生资源等领域循环产业链，提高循环利用水平。提高煤炭清洁利用水平，大力推广天然气、太阳能等清洁能源，争取生物质能源示范试点。切实加强污染整治。继续推进重点流域、重点区域、重点行业污染综合整治，加快污水垃圾处理、中水回用和污泥处理等设施建设，积极开展城市固体废弃物无害化处理，确保城乡空气、水质和声环境质量达到国家环境质量标准。继续抓好生态系统建设。积极实施农田防护林、特色经济林、林产工业原料林、森林抚育与改造等林业生态建设提升工程，完成植树造林8．3万亩，认真做好国家森林城市创建，争创省级生态县、生态乡镇和生态村。做好民权申甘林带、黄河故道湿地保护工作，发挥其文化内涵和生态、休闲旅游价值。加强林七、吴屯、任庄、郑阁水库等重点水源地的保护工作，确保城区居民饮水安全。抓好各类城市公园和生态绿地系统建设，努力建设生态商丘、美丽商丘、宜居商丘。

（八）着力保障和改善民生，实现发展成果人民共享。更加注重民生建设，解决人民群众最关心、最直接、最现实的利益问题，让发展成果最大程度地惠及人民群众，确保全年用于民生方面的支出占财政支出的比重不低于75％。推动实现高质量就业。继续实施更加积极的就业政策，同步推进政策扶持就业和鼓励创业带动就业，多渠道增加就业岗位，创新职业技能培训模式，切实做好高校毕业生、残疾人、低保对象及失业人员等特殊群体的就业工作。统筹推进社会保障体系建设。进一步完善城乡基本医疗、工伤、失业保险市级统筹、医疗保险门诊统筹等管理机制，巩固城乡居民养老保险试点成果。

扩大城镇职工基本养老保险、基本医疗保险和工伤保障覆盖面，着力解决跨区域制度衔接问题，完善失业保险与城市低保联动机制。严格落实被征地农民社会保障机制。加快社会服务体系建设。办好人民满意的教育，完善基本公共教育服务体系，启动二期学前教育三年行动计划，努力化解城镇“入园难”问题，推动义务教育均衡发展，实现高中教育、职业教育和高等教育全面发展。探索城乡教师互动交流机制，促进城乡教育资源共享。落实好促进民办教育发展政策，吸引社会资本投入教育事业。完善公共文化服务体系，统筹推进文化广电新闻出版事业，加大对公共文化基础设施建设投入，加强文化市场监管，创新文化体制机制，推动政府由办文化向管文化转变。实施文化产业“双十”工程，加快文化产业示范园区、文化改革发展试验区建设，继续做好大运河申遗工作。深入开展全民健身运动，扎实备战四年一次的省十二届运动会，开工建设市体育中心二期工程。深化医药卫生体制改革，完善基层医疗卫生服务体系，提升医疗服务水平。调整完善生育政策，促进人口长期均衡发展。落实价格调控政策，继续稳定价格总水平。提升社会治理能力。坚持源头治理、系统治理、综合治理、依法治理，努力提高社会治理水平，全面推进平安商丘建设，确保人民安居乐业、社会安定有序。做好社会矛盾排查化解，完善社会稳定风险评估机制，强化应急管理体系；畅通信访渠道，健全及时就地解决群众合理诉求机制，依法按政策解决群众反映强烈的信访问题；以基层基础为抓手，以技防建设为引领，构筑立体防控体系，强化社会治安综合治理，依法打击各类违法犯罪活动，提高群众的安全感和满意度；加强社区建设，健全基层服务平台和矛盾纠纷调解机制，做好社区矫正工作。加强安全生产和消防安全，完善制度和强化监管相结合，杜绝大的安全事故发生。强化食品药品安全监管，用最严格的标准、严密的监管、严厉的惩处、严肃的问责，确保人民群众“舌尖上的安全”！

培育和践行社会主义核心价值观，大力弘扬“一言九鼎、实干兴商”的商丘城市精神和商丘好人精神，把精神文明和道德建设融入国民教育、文明创建和社会建设的全过程，在全市培育做文明人、办文明事的良好风尚，形成体现我市优良传统和文化底蕴，营造适应发展要求的思想道德条件和文明社会环境。

加强国防教育和国防后备力量建设，支持驻商解放军、武警部队、预备役部队、民兵和人民防空建设，促进军民融合式发展。深入开展双拥共建，巩固发展军政军民团结。支持工会、共青团、妇联、工商联等人民团体发挥桥梁纽带作用。切实做好外事侨务、对台工作。继续做好民族宗教、统计、史志、气象、地震、社科研究等工作。扎实做好第三次经济普查。

继续办好事关人民切身利益的“十件实事”：1．扩大就业渠道。购买公益性岗位7000个，城镇新增就业6万人，下岗失业人员再就业2．1万人，投放5亿元小额贷款支持下岗失业人员、高校毕业生、就业困难人员创业。2．关心救助弱势群体。市级儿童福利院年内投入使用。完成9200名残疾人就业培训和630名0—6岁贫困残疾儿童抢救性康复工程。扩大法律援助覆盖面，受理案件4000件，实现应援尽援。3．切实维护公共安全。建设覆盖中心城区的社会治安视频监控系统，免费开展电动自行车打码登记服务，提升社会治安防控能力。4．改善城区交通状况。投资5000万元，完成11条背街小巷整修工程。投资1000万元，购买空调公交车，改善火车站至神火大道南端运营条件。5．实施文化惠民工程。开工建设豫东大剧院，实现博物馆免费对市民开放。完成“舞台艺术送农民”演出150场，“百戏进百乡”演出120场，农村电影放映4．6万场。6．改善城乡居民生活条件。投资2亿元，解决43万农村人口安全饮水问题。新建农村公路360公里。开工廉租住房和公共租赁住房15662套。投资4亿元，改造城区薄弱电网。投资2000万元，新建、改造14座联体式公厕垃圾中转站。7．推进生态环境治理。完成引黄调蓄日月湖主体工程。8．改善城区办学条件。在新区和梁园区、睢阳区新建、改扩建8所中小学校，逐步解决大班额问题。商丘幼师秋季开学投入使用。9．增强医疗卫生服务能力。新建新区人民医院（市妇幼保健中心和儿童医院）。10．提高社会保障水平。发放社会保障卡280万张。新型农村合作医疗人均筹资水平提高到380元，其中财政补助标准提高到每人每年320元，参合率98％以上。将企业退休人员基本养老金水平再提高10％。

四、以改革的精神推进政府自身建设

面对新形势、新任务和新要求，必须坚持从严治政、高效施政、依法行政、廉洁从政，积极打造为民、务实、清廉的公务员队伍，努力建设人民满意的政府。

（一）转变职能，推进政府服务创新。围绕市场在资源配置中起决定性作用和更好地发挥政府作用，实现政资分开、政企分开、政事分开和政社分开，进一步强化政府提供公共产品、搞好公共服务、维护社会公平正义的职能。认真落实国家、省简政放权各项措施，按照“能放尽放”的原则，继续取消和下放一批行政审批事项，切实简化办事程序，优化工作流程，规范服务标准，提高办事效率，提升服务水平，营造更加公平的市场环境。按照中央和省里的统一部署，有序推进食品药品监管、卫生和人口计划生育等部门职责和机构整合，全面完成政府机构改革任务。严控机构编制，确保财政供养人员只减不增。

（二）依法行政，推进政府科学决策。严格按照法定权限和程序行使权力、履行职责，依法管理经济和社会事务。认真执行人大及人大常委会的决定，自觉接受法律监督和工作监督，主动接受政协委员的民主监督，广泛听取各民主党派、人民团体和社会各界的意见和建议。高度重视人大代表建议和政协委员提案的办理工作，不断提高办理质量和水平。严格落实重大行政决策程序规定，把公众参与、专家咨询、风险评估、合法性审查和集体讨论决定，作为必经程序，确保科学决策和民主决策。拓宽政府信息公开、政务公开范围，认真落实预算公开制度，增强政府工作透明度，依法保障人民的知情权、参与权和监督权。支持审计、监察部门依法独立行使监督权。认真落实行政执法责任制和过错追究制，严格按照法定权限和程序行使权力、履行职责，不断提高依法行政能力和水平。

（三）务实重干，推进政府效能提升。以保持党同人民群众的血肉联系为重点加强作风建设，巩固“三治三提”和“两转两提”活动成果，扎实开展党的群众路线教育实践活动，着力解决“四风”方面存在的突出问题，深入实际、深入基层、深入群众，倾听群众呼声，关心群众疾苦，认真解决事关人民群众切身利益的问题。加强效能监察，整治庸懒散奢等不良风气，严肃查处推诿扯皮、行政不作为等行为。弘扬求真务实精神，坚持讲实话、办实事、求实效，精简文件、会议，改进文风、会风，把精力和重点放在转变发展方式、提高质量效益上，多做打基础、管长远、利全局的工作，健全完善科学发展考评体系，努力创造出经得起历史、实践和人民检验的政绩。

（四）廉洁从政，推进政府廉政建设。严格执行中央关于改进工作作风、密切联系群众的八项规定和省委市委20条贯彻落实意见。按照干部清正、政府清廉、政治清明的要求，加强政府机关工作人员廉洁从政教育和廉政文化建设。发扬艰苦奋斗优良作风，厉行节约，反对浪费，勤俭办事，把财力重点放在保障和改善民生上。推行廉政监督员制度，强化对政府机关及其工作人员，尤其是对窗口单位、关键部门和岗位的重点监督，坚决纠正损害群众利益的不正之风。严格执行党风廉政建设“一岗双责”，认真贯彻落实《廉政准则》，深入开展廉政建设和反腐败斗争，从决策和执行等环节加强对权力的监督，从源头上预防和治理腐败，营造风清气正的社会环境。

各位代表，我们面临的任务艰巨而繁重，我们肩负的责任重大而光荣！让我们紧密团结在以习近平同志为总书记的党中央周围，牢记使命，不负重托，上下一心，发奋图强，以更加振奋的精神、更加开阔的视野、更加务实的作风，为全力构筑中原经济区东部战略支撑做出新的贡献！
